# Multiple amino acid substitutions involved in the adaption of three avian-origin H7N9 influenza viruses in mice

**DOI:** 10.1186/s12985-018-1109-1

**Published:** 2019-01-08

**Authors:** Jianru Qin, Ouyang Peng, Xiaoting Shen, Lang Gong, Chunyi Xue, Yongchang Cao

**Affiliations:** State Key Laboratory of Biocontrol, School of Life Sciences, Higher Education Mega Center, Sun Yat-sen University, Guangzhou, 510006 China

**Keywords:** H7N9 influenza virus, virulence, adaption, mouse, amino acid substitution

## Abstract

**Background:**

Avian influenza A H7N9 virus has caused five outbreak waves of human infections in China since 2013 and posed a dual challenge to public health and poultry industry. The number of reported H7N9 virus human cases confirmed by laboratory has surpassed that of H5N1 virus. However, the mechanism for how H7N9 influenza virus overcomes host range barrier has not been clearly understood.

**Methods:**

To generate mouse-adapted H7N9 influenza viruses, we passaged three avian-origin H7N9 viruses in mice by lung-to-lung passages independently. Then, the characteristics between the parental and mouse-adapted H7N9 viruses was compared in the following aspects, including virulence in mice, tropism of different tissues, replication in MDCK cells and molecular mutations.

**Results:**

After ten passages in mice, MLD_50_ of the H7N9 viruses reduced >750-3,160,000 folds, and virus titers in MDCK cells increased 10-200 folds at 48 hours post-inoculation. Moreover, the mouse-adapted H7N9 viruses showed more expanded tissue tropism and more serious lung pathological lesions in mice. Further analysis of the amino acids changes revealed 10 amino acid substitutions located in PB2 (E627K), PB1 (W215R and D638G), PA (T97I), HA (H3 numbering: R220G, L226S, G279R and G493R) and NA (P3Q and R134I) proteins. Moreover, PB2 E627K substitution was shared by the three mouse-adapted viruses (two viruses belong to YRD lineage and one virus belongs to PRD lineage), and PA T97A substitution was shared by two mouse-adapted viruses (belong to YRD lineage).

**Conclusions:**

Our result indicated that the virulence in mice and virus titer in MDCK cells of H7N9 viruses significantly increased after adapted in mouse model. PB2 E627K and PA T97A substitutions are vital in mouse adaption and should be monitored during epidemiological study of H7N9 virus.

**Electronic supplementary material:**

The online version of this article (10.1186/s12985-018-1109-1) contains supplementary material, which is available to authorized users.

## Background

A novel reassortant avian influenza H7N9 virus was first detected in February 2013 and since then, it has posed an unprecedented threat to both public health and poultry industry [1, 2]. Until September 5, 2018, H7N9 influenza virus has caused 1,567 human infections, and 615 deaths, with a fatality rate of approximately 39% (World Health Organization, WHO). The number of laboratory confirmed human infections with H7N9 virus has surpassed that of H5N1 virus [3]. The novel H7N9 virus including avian-origin virus has been reported to be a tri-reassortant virus of H7, N9 and H9N2 influenza viruses [4, 5], and isolates from five epidemic waves of H7N9 influenza virus can be divided into many clades based on analysis of its genes [6–8]. However, numerous of new clades are continually occurring, indicated the continued evolution of H7N9 virus [9]. Moreover, highly pathogenic (HP) H7N9 virus were reported in late February 2017, which raised more concern about the pandemic threat of H7N9 virus [10, 11]. Herein, it is in urgent need to understand pathogenesis of H7N9 virus for control of this disease.

Though mouse is not a natural host of influenza viruses, it has been one of the most widely used animal models and has been applied to numerous areas of influenza research, including vaccine evaluation, virulence identification, virus adaptation, host-range comparison and so on [12–14]. Mice as an excellent animal model to study the mammalian adaptation of avian influenza viruses has been used for more than two decades [15–17]. A lot of previous work has convincingly proved that influenza virus could increase virulence in mice through lung-to-lung passage and many critical virulence-related sites were discovered by this method [18–20].

In this study, using sequential lung-to-lung passage in mice model, we adapted three H7N9 influenza viruses, A/Chicken/Guangdong/53/2014(H7N9) (H7N9-53), A/Chicken/Guangdong/MCX/2014(H7N9) (H7N9-MCX) and A/Chicken/Guangdong/ZSM/2017(H7N9) (H7N9-ZSM). Mouse-adapted H7N9 viruses were named H7N9-53 MA, H7N9-MCX MA and H7N9-ZSM MA, respectively. Evaluation of the virulence and replication feature of the six H7N9 viruses were conducted *in vivo* and *in vitro*. Moreover, sequencing and comparation of the whole genomes were performed to find out the amino acids that determine the increased virulence in the mouse.

## Methods

### Cells and viruses

Madin-Darby canine kidney (MDCK) cells were cultured in Dulbecco’s Modified Eagle’s Medium (DMEM, Gibco, NY) containing 10% fetal bovine serum (FBS, Gibco, NY) and used for assessment of H7N9 influenza virus replication.

A/Chicken/Guangdong/53/2014(H7N9) (H7N9-53), A/Chicken/Guangdong/MCX/2014(H7N9) (H7N9-MCX) and A/Chicken/Guangdong/ZSM/2017(H7N9) (H7N9-ZSM) were isolated from chickens in Guangdong, China and stored in our laboratory. H7N9-53 and H7N9-MCX belong to Yangtza River Delta (YRD) lineage and H7N9-ZSM belongs to Pearl River Delta (PRD) lineage.

### Adaption of the three H7N9 influenza viruses in mice

The mouse-adapted H7N9 variants were derived from independent series of sequential lung-to-lung passages of viruses in mice as described previously [21]. In brief, fifteen 6-week-old female BALB/C mice were randomly divided into three groups of five mice and inoculated intranasally (i.n.) with 50 μl of allantoic fluid containing the parental H7N9 viruses respectively. At 72 hours post-inoculation (hpi), lungs from the infected mice were harvested, homogenized and centrifuged. Supernatant was collected and inoculated to naïve mice at a volume of 50 μl for the next passage. After a total of 10 passages in mice, the three H7N9 variants in final lung homogenate were amplified by 10-day-old SPF chicken eggs for 72 h at 37 °C to prepare virus stocks. Mouse-adapted H7N9 viruses were designated as H7N9-53 MA, H7N9-MCX MA and H7N9-ZSM MA, respectively.

### EID_50_ and MLD_50_

Groups of 4-6 10-day-old specific pathogen-free (SPF) chicken eggs were inoculated with a series of ten-fold dilutions of the H7N9 viruses or supernatants of the homogenized organ samples at the amount of 0.1 ml. At 72 hpi, hemagglutination (HA) assay was performed to test HA titers in allantoic fluids. HA titer ≥ 4 log2 are defined as positive [22]. H7N9 viral titers in allantoic fluid and organ samples from infected mice were expressed as log_10_ EID_50_/ml. The detection limit of this assay was a titer of 0.75 log_10_ EID_50_/ml, and samples with titers less than 0.75 were assigned a value of 0.5.

The 50% mouse lethal dose (MLD_50_) of the six H7N9 viruses was performed as previously described [23]. Briefly, groups of five 6-week-old female BALB/C mice were inoculated intranasally (i.n.) with a series of ten-fold dilutions of the H7N9 virus at the volume of 50 μl, and mice inoculated with PBS was set as control. The mice were monitored daily for death for 14 days after inoculation and mouse that lost ≥ 25% body weight was humanely euthanized and regarded as dead. The values of EID_50_ and MLD_50_ were calculated by Reed–Muench method.

### Virulence comparation in mice

To compare the virulence of mouse-adapted H7N9 variants with parental H7N9 viruses *in vivo*, 6-week-old female BALB/C mice were inoculated intranasally (i.n.) with 10^6^ EID_50_ of the six H7N9 influenza viruses in 50 μl volume of PBS and monitored daily for weight loss and death for 14 days, respectively (n=5 per group). Moreover, mice inoculated with PBS was set as negative control. For determination of lung viral loads, three mice in each group were euthanized at 3 and 5 days post infection (dpi) and virus titers in lungs were determined by plaque assay. Furthermore, samples of hearts, liver, spleens, kidneys and brains were also collected at 3 dpi for determination of virus tissue tropism. Briefly, the organ samples were homogenized in 1 ml PBS and centrifuged at 10,000 × rpm for 10 min at 4°C, and the supernatant was used for test of EID_50_. Histopathological and immunohistochemistry (IHC) examination were also performed to identify lung lesions 3 dpi. In brief, lungs were fixed in neutral-buffered 10% formalin for 48 h, then embedded in paraffin. The paraffin-embedded lung tissues were sectioned at 4 μm and stained with haematoxylin and eosin (H&E) for examination under a light microscopy. Examination of influenza viral antigen in the lungs was performed by immunohistochemical analysis using an anti-influenza nucleoprotein (NP) antibody as previous described [24].

### Viral growth kinetics in MDCK cells

Viral growth kinetics in MDCK cells were used for comparison of mouse-adapted H7N9 variants and parental H7N9 viruses *in vitro*. MDCK cells were infected with H7N9 viruses at a multiplicity of infection (MOI) of 0.01, after incubation for one hour, MDCK cells were washed three times and overlaid with DMEM containing 1-2 μg/ml TPCK-treated trypsin. Supernatant was collected at 12, 24, 36, 48, 60 and 72 hpi. Virus titration in MDCK cells was determined by plaque assay and calculated by Reed–Muench method. Titers of virus were expressed as log_10_ PFU/ml.

### Sequence analysis

Viral RNA of the six H7N9 influenza viruses was extracted from the allantoic fluids using Trizol Reagent and reversed into cDNA by reverse transcription. Eight influenza viral gene segments were amplified by PCR as previously described [25] and sequenced by GENEWIZ biotechnology Co. Ltd. The results of sequencing were aligned by Lasergene sequence analysis software package (DNAStar, Madison, WI). The GeneBank accession numbers corresponding to H7N9-53 virus are MH553113-MH553119 and KY221841; H7N9-MCX are MH553124-MH553130 and KY221844; H7N9-ZSM are MH553137-MH553144 (Additional file 1: Table S1).

### Statistical analysis

Statistics analysis were performed using GraphPad Prism 6. Unpaired Student's t-tests or ANOVA followed by Dunnett’s multiple comparison tests were used for statistical comparisons and statistics analysis. Statistical difference between two groups was indicated by * (*p*<0.05), ** (*p*<0.01), *** (*p*<0.001) and **** (*p*<0.0001).

## Results

### Adaption of H7N9 influenza viruses to mice

Firstly, the pathogenicity of parental H7N9-53, H7N9-MCX and H7N9-ZSM were evaluated in mice, and the three H7N9 viruses were unlethal to mice even at a high dose of 10^8.0^ EID_50_ (Table 1). In order to generate mouse-adapted variants (designated as H7N9-53 MA, H7N9-MCX MA and H7N9-ZSM MA, respectively), serial lung-to-lung passages of the three H7N9 viruses were performed in mice independently. After 10 passages, MLD_50_ of H7N9-53 MA, H7N9-MCX MA and H7N9-ZSM MA were 4.32, 5.12 and 1.50 log_10_ EID_50_/ml, respectively (Table 1). Compared to the parental H7N9 viruses, MLD_50_ of the mouse-adapted variants decreased 10^2.88^-10^6.50^ folds. These results indicated that the virulence of the three H7N9 influenza viruses markedly increased in mice through serial passages.

**Table 1.** MLD_50_ of the parental and mouse-adapted H7N9 viruses

Influenza virus	MLD_50_	Decrease in MLD_50_
	(log_10_ EID_50_/ml)	(log_10_ EID_50_/ml)
H7N9-53	>8.0	-
H7N9-53 MA	4.32	>3.68
H7N9-MCX	>8.0	-
H7N9-MCX MA	5.12	>2.88
H7N9-ZSM	>8.0	-
H7N9-ZSM MA	1.50	>6.5

-: Not applicable

### Pathogenicity feature and tissue tropism of H7N9 viruses in mice

To compare the virulence of the parental and mouse-adapted H7N9 viruses *in vivo*, mice were inoculated intranasally (i.n.) with 10^6^ EID_50_ of each virus and a series of assays including body changes, survival rates, virus titers in different tissues, histopathological and immunohistochemistry examination were carried out.

**Fig. 1.**
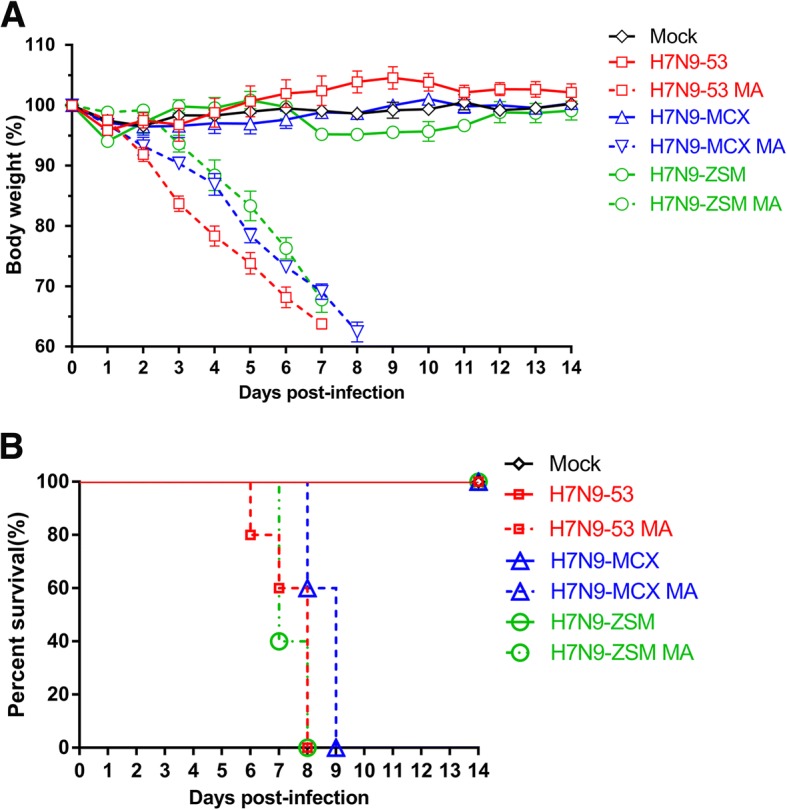
Pathogenicity of the parental and mouse-adapted H7N9 viruses in mice. Groups of 5 6-week-old female BALB/C mice were inoculated i.n. with 10^6^ EID_50_ (50 μl) of H7N9-53, H7N9-53 MA, H7N9-MCX, H7N9-MCX MA, H7N9-ZSM, H7N9-ZSM MA and PBS, respectively. Body weight changes and survival rates were monitored daily for 14 days. **a** Weight changes were recorded daily and represented by means (± standard deviation). **b** Mortality was determined by percentage of the surviving mice.

Mice infected with the parental H7N9 influenza virus survived 14 days with a slight weight loss, but they recovered to normal body weight soon (H7N9-ZSM recovered to normal body weight at 12 dpi, and mice inoculated with it were the latest recovered) (Fig. 1a, b). In contrast, mice infected with the mouse-adapted variants lost body weight from 2-3 dpi (Fig. 1a), and they showed severe post-infection symptoms, such as mental depression, severe emaciation, lumbar back arch, loss of appetite, ruffled fur and all succumbed to infection at 8 dpi (Fig. 1b).

**Fig. 2.**
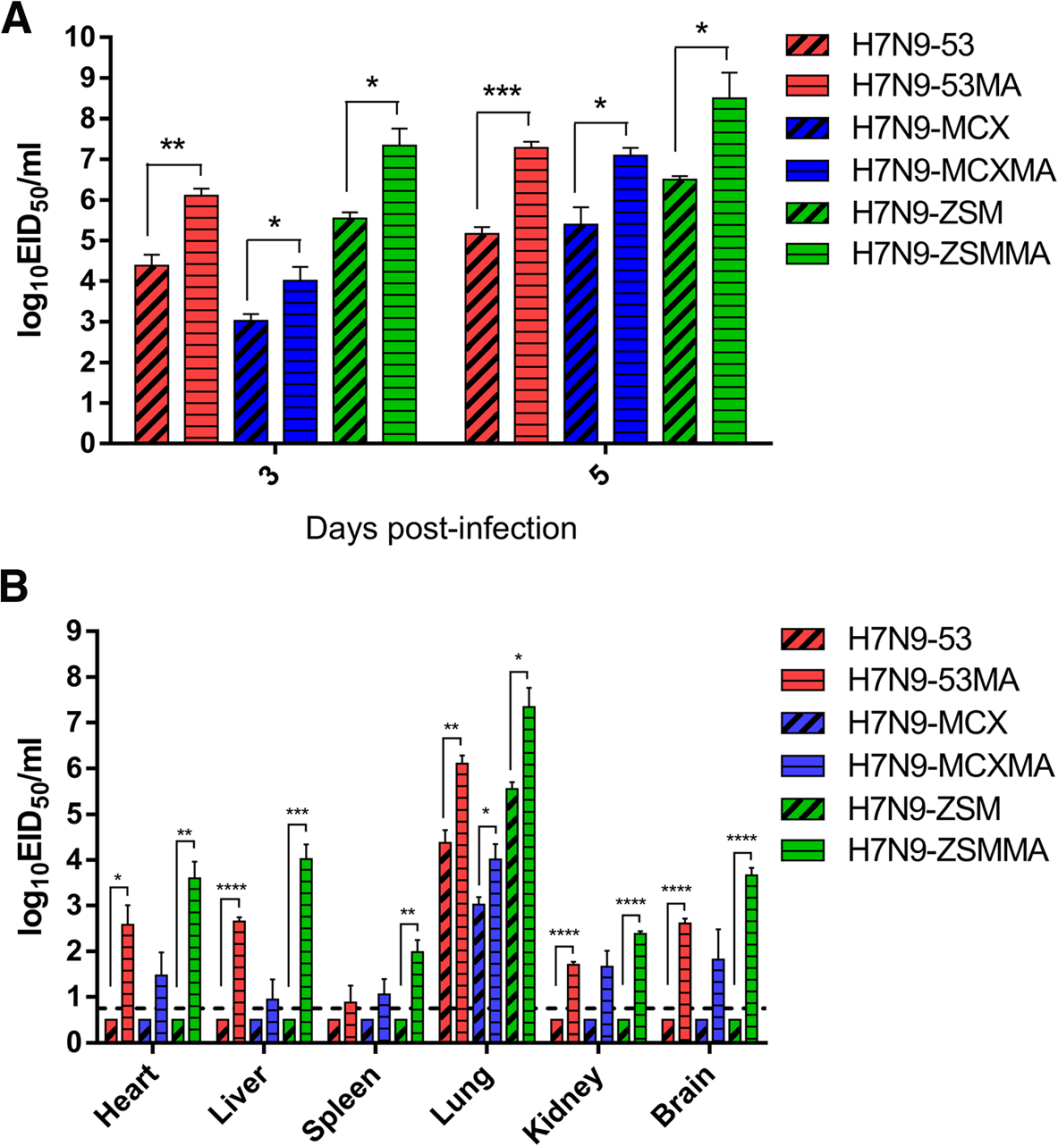
Titers of virus in different tissues of the parental and mouse-adapted H7N9 viruses in mice. Groups of 6 6-week-old female BALB/C mice were inoculated i.n. with 10^6^ EID_50_ (50 μl) of H7N9-53, H7N9-53 MA, H7N9-MCX, H7N9-MCX MA, H7N9-ZSM, H7N9-ZSM MA and PBS, respectively. 3 dpi, 3 mice were euthanized to collect hearts, liver, spleens, lungs, kidneys and brains for determination of virus titers by EID_50_. The remaining 3 mice were euthanized for lung viral loads by 5 dpi. **a** Lung viral loads of mice inoculated with H7N9 virus at 3 and 5 dpi. **b** Viral distribution of H7N9 virus in different tissues. The limit of virus detection is indicated by a dotted line.

Lung viral loads were determined 3 and 5 days after inoculation with each H7N9 viruses and the results showed lung viral titers of the mouse-adapted variants were significantly higher than that of their parental viruses no matter at 3 dpi (*p*=0.0061-0.0491) or at 5 dpi (*p*=0.0007-0.0360) (Fig. 2a). Moreover, Lung viral titers at 5 dpi were higher than at 3 dpi demonstrated constant replication of viruses in lungs (Fig. 2a). The lung viral titers of H7N9-53 vs H7N9-53 MA, H7N9-MCX vs H7N9-MCX MA, H7N9-ZSM vs H7N9-ZSM MA were 4.38 ± 0.39 vs 6.11 ± 0.24, 3.02 ± 0.23 vs 4.01 ± 0.41, 5.55 ± 0.21 vs 7.34 ± 0.60 at 3 dpi and 5.16 ± 0.24 vs 7.29 ± 0.21, 5.39 ± 0.61 vs 7.1 ± 0.27, 6.51 ± 0.11 vs 8.50 ± 0.90 at 5dpi. Taken together, the three mouse-adapted H7N9 variants replicated more effectively in mice lungs compared to their parental H7N9 viruses.

Furthermore, virus titers in heart, liver, spleen, lung, kidney and brain were tested for comparation of tissue tropism between the parental and mouse-adapted H7N9 viruses at 3 dpi. H7N9 mouse-adapted variants could be detected in all tissues, but H7N9 parental viruses could not be detected in any tissues except of lungs (Fig. 2b). Virus titers in tissues from the mouse-adapted H7N9 variants revealed that lungs were the highest, followed by livers, hearts, brains, virus titers in spleens and kidneys were the lowest. All, H7N9-53 MA, H7N9-MCX MA and H7N9-ZSM MA had more expanded tissue tropism in mice than H7N9-53, H7N9-MCX and H7N9-ZSM (Fig. 2b).

**Fig. 3.**
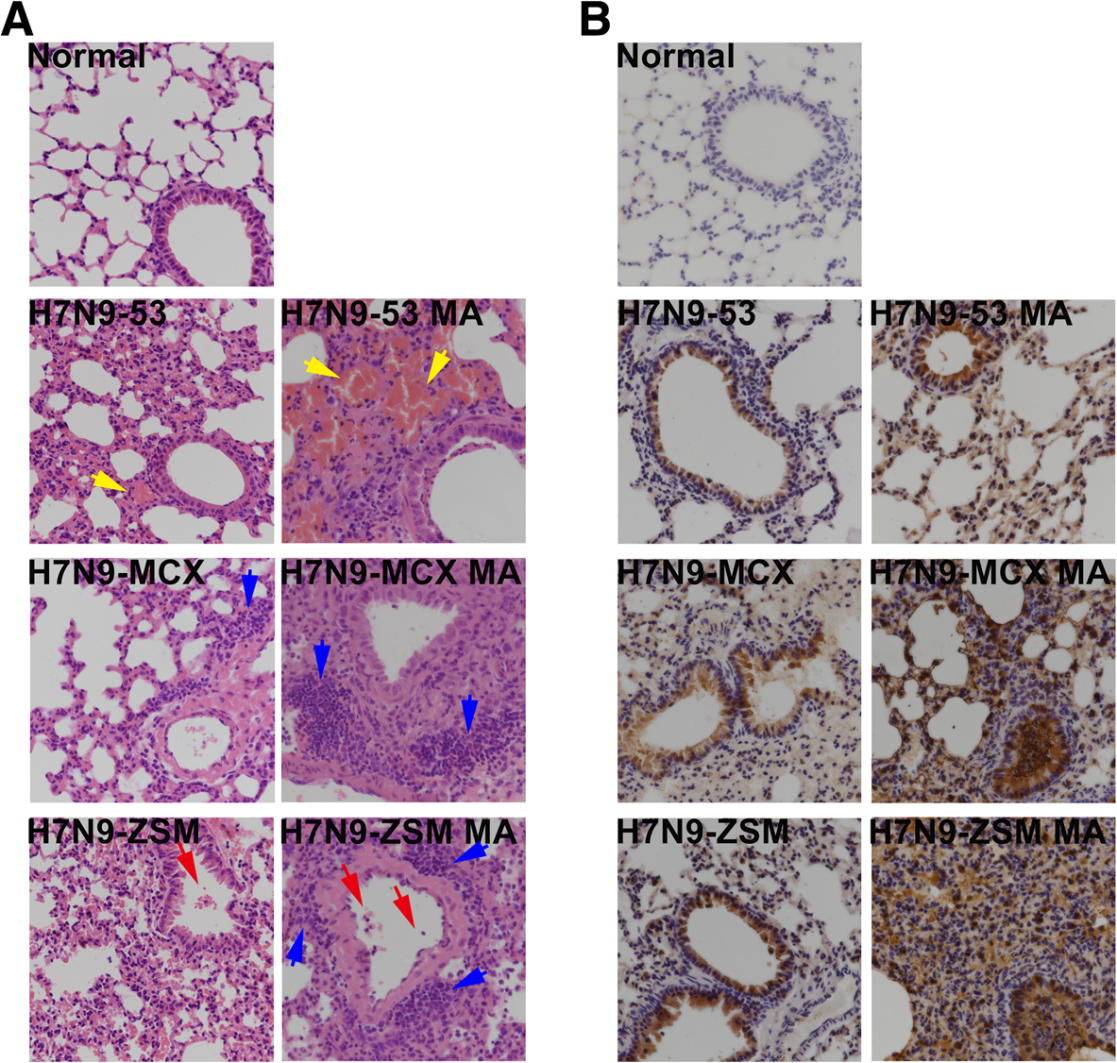
Histopathology and immunohistochemistry examination of lungs of the parental and mouse-adapted H7N9 viruses. 3 days after the inoculation, the lungs (*n* = 3) were examined by H&E staining for pathological changes and by anti-influenza nucleoprotein (NP) antibody for detection of antigens (×400). **a** Histopathology examination. Yellow, blue and red arrows indicated congestion, inflammatory cells infiltration and deciduous cells in the bronchial lumen, respectively. **b** Immunohistochemistry examination.

Histopathological examination of lungs revealed that mice inoculated with the mouse-adapted H7N9 variants showed severe lesions with congestion, inflammatory cells infiltration or deciduous cells in the bronchial lumen. However, lesions in lungs from mice inoculated with the parental H7N9 viruses were relatively mild (Fig. 3a). Viral detection in lungs by IHC showed that positive stained cells were widely distributed in the mouse-adapted H7N9 variants groups, indicating lung viral loads were high. Positive signals were relatively weak in mice lungs from the parental H7N9 viruses (Fig. 3b). Neither lesion nor H7N9 virus antigen was observed in lungs from mock mice as expected (Fig. 3).

### Growth characteristics of H7N9 viruses in MDCK cells

**Fig. 4.**
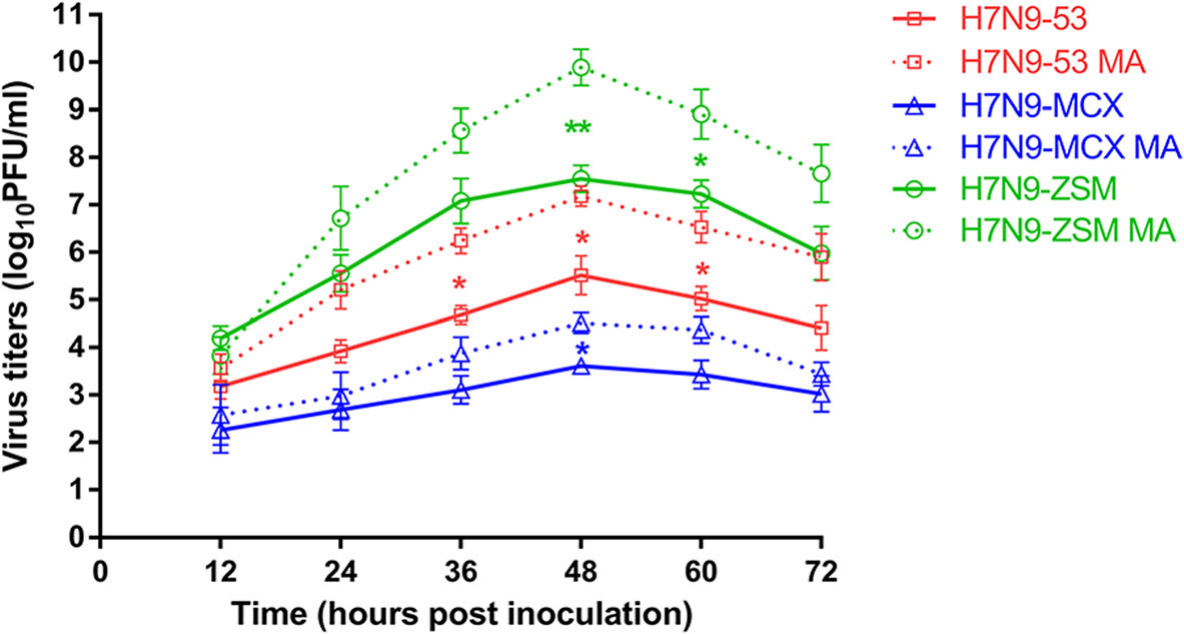
Growth kinetics of the parental and mouse-adapted H7N9 viruses. MDCK cells were inoculated with H7N9-53, H7N9-53 MA, H7N9-MCX, H7N9-MCX MA, H7N9-ZSM, H7N9-ZSM MA (MOI=0.01), respectively. Supernatants of the cultured cells were collected at a 12-hour interval till 72 hpi. Subsequently, the virus titers were measured by plaque assay and represented by means (± standard deviation).

Comparation of replication ability *in vitro* between the parental and mouse-adapted H7N9 viruses was also conducted in MDCK cells. Growth kinetics revealed that compared to their parental H7N9 viruses, the mouse-adapted H7N9 viruses grew faster and achieved to higher titers (Fig. 4). All the six H7N9 viruses reached the highest virus titers at 48 hpi, and the mean titers of H7N9-53, H7N9-53 MA, H7N9-MCX, H7N9-MCX MA, H7N9-ZSM and H7N9-ZSM MA were 5.52, 7.18, 3.52, 4.52, 7.55 and 9.89 log_10_PFU/ml, respectively (*p*=0.0078-0.0296) (Fig. 4). These results indicated that the increased virulence in mice of the mouse-adapted viruses accompanied by the enhanced replication ability *in vitro*.

### Sequence analysis

To further explore the molecular changes of mouse-adapted H7N9 viruses which increase the virulence in mice, genomes of the six H7N9 viruses were sequenced. The results revealed that there was a total of 13 amino acid differences located in 10 sites of influenza virus genomes between the parental and mouse-adapted H7N9 viruses, of which 5 between H7N9-53 and H7N9-53 MA, 5 between H7N9-MCX and H7N9-MCX MA, 3 between H7N9-ZSM and H7N9-ZSM MA, respectively. Moreover, E627K substitution in PB2 protein was the common substitution of the three mouse-adapted H7N9 viruses and PA T97I substitution was shared by H7N9-53 MA H7N9-ZSM MA. The amino acid substitutions were mapped in PB2, PB1, PA, HA and NA proteins (Table 2), and genes of NP, M and NS were 100% homologous.

**Table 2.** Amino acid differences between the parental and mouse-adapted H7N9 viruses

Influenza virus	PB2	PB1	PA	HA		NP	NA	M	NS
	627	-	97	235^a^ (226^b^)	288^a^ (279^b^)	-	134	-	-
H7N9-53	E	-	T	L	G	-	R	-	-
H7N9-53 MA	K	-	I	S	R	-	I	-	-
	627	215	97	229^a^ (220^b^)		-	3	-	-
H7N9-MCX	E	W	T	R		-	P	-	-
H7N9-MCX MA	K	R	I	G		-	Q	-	-
	627	638	-	503^a^ (493^b^)		-	-	-	-
H7N9-ZSM	E	D	-	G		-	-	-	-
H7N9-ZSM MA	K	G	-	R		-	-	-	-

**-**: Not applicable

^a^: H7 numbering

^b^: H3 numbering

In addition, we queried H7N9 sequences deposited in the GISAID EpiFlu database, and the number of H7N9 viruses with amino acid substitutions identified in this study were also displayed in the Table 3.

**Table 3.** Count the number of H7N9 viruses with amino acid substitutions identified in this study

Segment	Position	Amino acid	Frequency of substitution (no. of strains with the substitution/total no. of strains)
PB2	627	E	1351/2370
		K	915/2370
PB1	215	W	1/2358
		R	2007/2358
	638	D	2197/2358
		G	41/2358
PA	97	T	2342/2357
		I	7/2357
HA	229	R	2619/2647
		G	1/2647
	235	L	2590/2647
		S	10/2647
	288	G	2276/2647
		R	7/2647
	503	G	12/2647
		R	0/2647
NA	3	P	2586/2620
		Q	1/2620
	134	R	2337/2620
		I	0/2620

## Discussion

Human infections of H7N9 virus have spread from mainland China to Hong Kong and Taiwan, even to Canada and Malaysia, causing unprecedented losses to public health (WHO). Though H7N9 virus does not poss sufficient ability for human-to-human transmission, it could effectively replicate in alveolar epithelial cells of mammals and transmit via respiratory droplets to kill ferrets [14], so it is hard to predict whether this disease will cause a pandemic in the future. H7N9 virus has generated multiple genotypes for continuous evolution [26]. In general, most of the isolates can be clustered to two lineages (YRD lineage and PRD lineage). YRD lineage includes Zhejiang, Jiangsu, Shanghai, and PRD lineage refers to Guangdong, Guangxi, Fujian, Hongkong [27]. Viruses of the YRD lineage reacted poor with ferret antiserum raised by the PRD candidate vaccine (WHO). Herein, it is important to monitor the prevalence of H7N9 virus belonging to both YRD and PRD lineage. In this report, H7N9-53 and H7N9-MCX belong to YRD lineage, and H7N9-ZSM belongs to PRD lineage. Through adaption in mice of the three H7N9 influenza viruses, we tried to seek virulence-associated mutations, as well as compare mouse-adapted substitutions between YPD and PRD lineages.

There are many factors influence on mammal adaption of avian influenza virus and the most important determinant is receptor-binding specificity [28]. As is known to all, the first step of influenza virus infect host cells is binding to sialyloligosaccharides on cell surface via hemagglutinin (HA) protein [29]. Avian influenza virus HAs prefer binding to α2,3-linked sialic acids, whereas HAs of human influenza virus prefer α2,6-linked sialic acids [30]. Different sialic acid receptor-binding properties make host-range and change may result in crossing of host barrier [31]. Another major determinant of host-range is polymerase activity. Compared with temperature of mammalian upper respiratory tract, temperature of avian gastrointestinal tract is much higher (38°C of avian vs 33°C of mammalian). Amino acid substitutions in polymerase protein usually lead to changing of viral replication efficiency in different respiratory tract [32, 33]. Moreover, N-linked glycosylation of HA protein is also an important factor influence on host-range of influenza virus. Glycosylation plays an important role in HA protein folding and HA antigenicity, and changes at glycosylation sites possible produce improperly folded HA proteins and then affect the antigenicity of HA [34]. In addition to the above-mentioned factors, several determinants also contribute to mammal adaption, such as morphology of influenza virus, acid stability of HA protein and functional balance between HA and NA [35]. In this study, a total of 9 amino differences was discovered between the parental and mouse-adapted H7N9 viruses. Among the substitutions, 4 located in HA proteins, 2 in NA protein and 4 in polymerase proteins (1 in PB2, 2 in PB1 and 1 in PA protein).

Further comparation of the amino acids substitutions discovered that only E627K at PB2 protein shared by the three mouse-adapted H7N9 viruses. PB2 subunit has multiple domains and 627-nuclear localization signal (NLS) domain is the main region that involved in interaction with NP protein [36]. Moreover, PB2 627-NLS domain has been proved to play the role in transcription and replication of influenza virus RNA genome [37]. Many subtypes of influenza virus, including H3N2, H5N1, H5N5, H5N6, H6N1, H6N6, H7N1, H7N7, H7N9 and H9N2 have been reported increasing virulence by substitution of E627K at PB2 protein [38–40]. Moreover, the evaluation of H7N9 viruses isolated from avian species between 2013 and 2017 in China revealed some H7N9 viruses have readily obtained the 627K mutation in its PB2 segment upon replication in ferrets, causing it to become highly lethal in mice and ferrets [5]. In addition to the most often-observed substitution of E627K, there are several other important mutations in the PB2 protein, such as E158G, D253N, T271A, K526R, Q591K, A588V, D701N and so on, and these mutations had been proved to enhance polymerase activity [5, 16, 19, 20, 32]. Another substitution worth noting is T97I at PA protein. This substitution is shared by the two H7N9 viruses belonging to PRD lineage. PA T97I substitution was reported in mouse adaption of H5N2, H6N1, H7N1, H10N7 and H7N9 subtypes of influenza viruses [13, 41–47]. But much work needs to be done about the validation and mechanism. In addition, other substitutions discovered in this study have not been reported, indicating that these mutations are a specific selection of H7N9 virus.

One thing needs to be noted is that though 10 successive passages in mice were performed to obtain three mouse-adapted H7N9 influenza viruses, the pathogenicity to mice and replication in cells of the three mouse-adapted H7N9 viruses were different. The MLD_50_ of H7N9-ZSM MA is 660 and 4,164 times less than that of H7N9-53 MA and H7N9-MCX MA, respectively. Virus titer in MDCK of H7N9-ZSMMA was the highest, followed by H7N9-53 MA, H7N9-MCX MA was the lowest. All these findings demonstrated that though E627K substitution is the common mutation of the three mouse-adapted viruses and it has been proved to increase virulence in mice, it is not the only virulence-determination mutation, there must be other substitutions correlate with E627K to influence virulence of mouse-adapted H7N9 virus.

## Conclusion

The virulence and replicative ability of three H7N9 influenza viruses increased through the sequential lung-to-lung passages, and several mutations were found, which may influence on the virulence and growth characteristics of H7N9 viruses. Moreover, PB2 E627K and PA T97I may play important roles in H7N9 mammal adaption, but the exact role and other substitutions need further verification. Our study attaches great importance to the epidemiological surveillance of H7N9 virus.

## Additional files

Additional file 1: **Table S1.** GeneBank accession numbers corresponding to the three H7N9 viruses. (DOCX 15 kb)

## Abbreviations

WHO: World Health Organization\
HP: Highly Pathogenic
MDCK: Madin-Darby canine kidney
DMEM: Dulbecco’s Modified Eagle’s Medium
FBS: Fetal bovine serum
H7N9-53: A/Chicken/Guangdong/53/2014(H7N9)
H7N9-MCX: A/Chicken/Guangdong/MCX/2014(H7N9)
H7N9-ZSM: A/Chicken/Guangdong/ZSM/2017(H7N9)
YRD: Yangtza River Delta
PRD: Pearl River Delta
SPF: Specific pathogen-free
HA: Hemagglutinin
EID_50_: 50% egg infectious dose
MLD_50_: 50% mouse lethal dose
i.n.: Inoculated intranasally
dpi: days post infection
IHC: Immunohistochemistry
H&E: Haematoxylin and eosin
NP: Nucleoprotein
MOI: Multiplicity of infection
hpi: Hours post inoculation.
