# Supplementary material for: Multiple amino acid substitutions involved in the adaption of three avian-origin H7N9 influenza viruses in mice
Source: Virol J. 2019 Jan 8;16:3. doi: 10.1186/s12985-018-1109-1 (PMC6323857; doi:10.1186/s12985-018-1109-1)
Supplement: Supplementary file 1 — Table S1. GeneBank accession numbers corresponding to the three H7N9 viruses. (DOCX 15 kb) [file 12985_2018_1109_MOESM1_ESM.docx]

**Additional file 1: table S1.** GeneBank accession numbers corresponding to the three H7N9 viruses.

| **Segment** | **H7N9-53** | **H7N9-MCX** | **H7N9-ZSM** |
| --- | --- | --- | --- |
| **PB2** | MH553113 | MH553124 | MH553137 |
| **PB1** | MH553114 | MH553125 | MH553138 |
| **PA** | MH553115 | MH553126 | MH553139 |
| **HA** | KY221841 | KY221844 | MH553140 |
| **NP** | MH553116 | MH553127 | MH553141 |
| **NA** | MH553117 | MH553128 | MH553142 |
| **M** | MH553118 | MH553129 | MH553143 |
| **NS** | MH553119 | MH553130 | MH553144 |
